# Association Between Gut Microbiome Composition and Physical Characteristics in Patients with Severe Motor and Intellectual Disabilities: Perspectives from Microbial Diversity

**DOI:** 10.3390/nu16203546

**Published:** 2024-10-19

**Authors:** Suzumi Kageyama, Rikako Inoue, Koji Hosomi, Jonguk Park, Hitomi Yumioka, Miki Doi, Miyuu Miyake, Yuka Nagashio, Yoshiko Shibuya, Nobue Oka, Hirofumi Akazawa, Susumu Kanzaki, Kenji Mizuguchi, Jun Kunisawa, Yasuyuki Irie

**Affiliations:** 1 Graduate School of Health and Welfare Science, Okayama Prefectural University, Okayama 719-1197, Japan; suzumi.kageyama@gmail.com (S.K.); doimiki1214@gmail.com (M.D.); miyuu.20002690@gmail.com (M.M.); yuka.nagashio@gmail.com (Y.N.); 2 Research Fellow of Japan Society for the Promotion of Science, Tokyo 102-0083, Japan; 3 Department of Nutritional Science, Faculty of Health and Welfare Science, Okayama Prefectural University, Okayama 719-1197, Japan; rinoue@fhw.oka-pu.ac.jp; 4 Microbial Research Center for Health and Medicine, National Institutes of Biomedical Innovation, Health and Nutrition (NIBIOHN), Osaka 567-0085, Japan; hosomi@omu.ac.jp (K.H.); yumioka@osaka-seikei.ac.jp (H.Y.); kunisawa@nibiohn.go.jp (J.K.); 5 Graduate School of Veterinary Science, Osaka Metropolitan University, Osaka 598-0048, Japan; 6 Artificial Intelligence Center for Health and Biomedical Research, National Institutes of Biomedical Innovation, Health and Nutrition (NIBIOHN), Osaka 567-0085, Japan; jonguk@nibiohn.go.jp (J.P.); kenji@protein.osaka-u.ac.jp (K.M.); 7 Faculty of Nutrition, Osaka Seikei College, Osaka 533-0007, Japan; 8 Department of Pediatrics, Asahigawasou Rehabilitation and Medical Center, Okayama 703-8207, Japan; kango@jidouin.jp (Y.S.); sunflower.nopi@gmail.com (N.O.); hiro23@fa2.so-net.ne.jp (H.A.); kanzaki@asahigawasou.or.jp (S.K.); 9 Institute for Protein Research, Osaka University, Osaka 565-0871, Japan

**Keywords:** severe motor and intellectual disabilities, gut microbiome, maturation, fecal metabolites, bile acids

## Abstract

Background: The human gut environment undergoes substantial changes as a host ages. This investigation centered on the gut microbiome diversity among patients with severe motor and intellectual disabilities (SMID), examining the association between the gut microbiome composition and physical characteristics with varying levels of diversity. Methods: Fourteen subjects were investigated, with physical and defecation status, blood biochemical test, gut microbiome profiling, and fecal metabolites used to divide the patients into a high-diversity group (HD, eight patients) and a low-diversity group (LD, six patients). Results: Findings indicated that the microbiome of the LD group showed delayed maturation reminiscent of neonates and lactating infants. Analysis of the fecal bile acids (BAs) revealed a markedly diminished proportion of deoxycholic acid in the secondary BAs in the LD group, suggestive of inadequate conversion from primary to secondary BAs. Furthermore, the LD group presented with loose stools. The LD group exhibited a higher degree of physical severity, with all patients bedridden and fed via gastrostomy with only enteral formula received. Conclusions: The composition of the gut microbiome and BAs in the LD group was found to differ from those of healthy individuals and the HD group, indicating a potentially immature gut environment for these individuals.

## 1. Introduction

Patients with severe motor and intellectual disabilities (SMID) often have multiple health problems that require ongoing medical management. As many as 35 different symptoms have been reported, including epilepsy and pulmonary and respiratory diseases [1]. Among these, constipation is a frequent comorbidity [2] and a critical issue because it can lead to gastroesophageal reflux disease or death due to intestinal obstruction [3,4]. We, therefore, conducted a pilot study of 10 SMID patients (15.5 (11, 28) years old) receiving care and classified the groups based on similarities in the gut microbiome profiles [5]. The results showed that differences in gut microbiome composition in SMID patients were associated with differences in physical severity and constipation symptoms, while also indicating that SMID patients with more severe constipation symptoms and a lower-diversity gut microbiome, with higher genus *Bifidobacterium* and lower order *Clostridiales*, tended to be bedridden, tube fed, or taking muscle relaxants.

The human gut microbiome undergoes substantial compositional changes throughout the lifespan, profoundly impacting human health. Distinct variations in the gut microbiome composition have been observed across the different age groups: infants, young children, adults, and the elderly [6], with particularly noteworthy shifts observed in the gut bacterial community composition during the transition from infancy to a more diversified diet [7]. Research indicates that by approximately three years of age, the diversity and composition of the gut microbiome closely resemble that of adults [8]. One study on Japanese subjects spanning various age groups revealed continuous alterations in bacteria such as *Bifidobacterium* and *Lachnospiraceae* (family level in the order *Clostridiales*), as well as changes in the alpha diversity, which was particularly noticeable in individuals under 20 years old, reflecting the process from an immature to a mature human gut microbiome [7]. Hence, diversity serves as a potential indicator of gut microbiome maturation.

Metabolites such as short-chain fatty acids (SCFAs) and bile acids (BAs), which are produced by intestinal bacteria, play a critical role in maintaining host homeostasis. SCFAs are generated through the fermentation of dietary fiber by gut bacteria while also serving as an energy source for the host and are pivotal in regulating the energy metabolism of the host as receptor-mediated signal transducers [9]. SCFAs also stimulate colonic smooth muscle and are involved in intestinal motility, which has been reported to be associated with constipation [10].

Primary BAs are synthesized in the liver, secreted into the intestinal tract for fat absorption, and predominantly reabsorbed to circulate throughout the body. Unabsorbed BAs are metabolized by gut bacteria into secondary BAs, which have been reported to strongly activate G-protein coupled bile acid receptor 1 (also known as TGR5), which is involved in a wide range of physiological functions including glucose and lipid metabolism, energy homeostasis, and regulation of the immune response [11]. The involvement of TGR5 in intestinal motility and water secretion has been reported to be associated with constipation [10]. Notably, BA metabolism in the intestine has been observed to undergo progressive changes that correspond with the maturation of the gut microbiome [11]. Primary BAs dominate prior to weaning; however, as weaning progresses and the gut microbiome matures, the occupancy of secondary BAs increases. Dehydroxylation in secondary BA metabolism has been linked to microbial diversity [11], which is, in turn, associated with BA composition.

The gut microbiota has also been reported to be associated with malnutrition in children while also contributing to the proliferation and maturation of intestinal epithelial cells, induction of host genes for nutrient uptake, and development of the mucosal immune system [12], all of which are important for optimal nutrient absorption. Secondary analysis of cohort studies investigating twins in Malawi and Bangladesh reported that the severity of stunting is associated with reduced gut microbiome diversity [13].

Our pilot study [5] indicated that some patients with SMID had a low-diversity gut microbiome. Another study comparing the gut microbiome of SMID patients on tube feeding to healthy subjects found that these patients had lower microbiome diversity and exhibited a distinct microbial composition [14], suggesting that some SMID patients may have reduced gut microbiome diversity. However, research into the gut microbiota of SMID patients remains limited, particularly with regard to the use of next-generation sequencing technologies, and the specific characteristics of SMID patients with low diversity have not been thoroughly explored. Few studies have integrated gut microbiome analysis with fecal metabolites. The results of our pilot study [5] also indicated that five of six SMID patients under the age of 18 were severely stunted in terms of height for age (H/A), indicating stunting, with the sixth patient also demonstrating moderate stunting. However, few studies on the gut microbiome of patients with SMID have integrated nutritional evaluation, such as blood biochemical tests. This study focuses on the diversity of the gut microbiome in SMID patients with the aim of elucidating the relationship between gut microbiome composition and the physical characteristics observed in these patients, in conjunction with fecal metabolites such as SCFAs and BAs, and nutritional evaluation including blood biochemical tests for markers such as rapid turnover proteins (RTPs).

## 2. Materials and Methods

### 2.1. Participants

Consent for inclusion in this cross-sectional study was obtained from 14 hospitalized patients aged over 3 and under 18 years (male: eight; female: six) at a residential facility for patients with disabilities in Okayama, Japan. Patients were included under the following criteria: (1) receiving medical subsidies for SMID, based on diagnosis by a physician, and (2) Oshima’s classification [15] of 1–9 indicating that a patient is bedridden, can sit only with support, or suffers from gait disturbance with an IQ less than 50. Patients who had consumed antibiotics within one month were excluded. The protocol used in this study was approved by the Ethics Committee of Okayama Prefectural University (Approval No. 18-70) and registered in the clinical trial registration system (UMIN000043009) in accordance with the Declaration of Helsinki.

### 2.2. Survey Details and Fecal Sample Collection

Height and weight measurements and a survey of weaning food experiences were conducted by facility nurses. Height for age (H/A) and weight for height (W/H) were used to determine nutritional status, with the average height and weight for age and gender in the 2000 infant physical growth survey from the Ministry of Health, Labor, and Welfare and the 2000 school health statistics survey from the Ministry of Education, Culture, Sports, Science, and Technology utilized to obtain the necessary information. Nutrient intake was determined by the menu provided in the week prior to the survey in the inpatient facility, and the amount of nutrients calculated using nutrition calculation software V9EX Eiyokun (Kenpakusha, Tokyo, Japan), with the equivalent daily consumption calculated from the intake records. The nutrition of tube-fed patients was identified from the nutrient composition of each enteral formula. The Japanese version of the Constipation Assessment Scale (CAS) [16] and the Bristol Stool Scale (BSS) [17] were used to investigate the defecation status, and the CAS evaluation standard was used to calculate the percentage of evaluation points according to the number of items for each patient and evaluate the presence of constipation at percentages exceeding 31.3% [5,18], corresponding to the evaluation standard (5 points out of 16) adopted in a previous study [16]. Prescription drugs were classified into 15 categories based on the indications listed on the package insert and included gastric acid secretion inhibitors and antibiotics with small-dose long-term macrolide therapy, which have been reported to affect the gut microbiome [19,20]. Patients in Japan with SMID commonly take multiple drugs for multiple conditions [21,22]. Therefore, no restrictions were placed on the use of prescription drugs, other than short-term antibiotics, when examining the general status of patients with SMID. In addition, the antimicrobial agent sulfamethoxazole–trimethoprim (ST) combination is also used for small-dose long-term prophylactic therapy. It has been reported that small-dose long-term therapy with ST combination in children may selectively suppress the growth of *o_Enterobacteriales* [23]; however, the previous study examining the gut microbiome of patients with SMID have also included those taking ST combination [24]. For this reason, this study did not exclude patients who had been receiving small-dose long-term ST combination therapy, and analyzed the ST combination drug by adding it to the antibiotic category. For blood biochemical testing, samples were collected by the facility nurses at least two hours after breakfast. Two patients did not give consent for blood collection. Quantitative analysis was outsourced to the hematology laboratory in the inpatient facility. 

Fecal samples from each patient were collected in guanidine thiocyanate solution (TechnoSuruga Laboratory, Shizuoka, Japan) and stored at 4 °C until DNA extraction for 16S rRNA gene amplicon sequencing. Another set of fecal samples was stored at −80 °C for the measurement of fecal metabolites (BAs and SCFAs).

### 2.3. BA Measurement

BA measurements were outsourced to TechnoSuruga Laboratory Co., Ltd. (Shizuoka, Japan) and analyzed using a liquid chromatograph–quadrupole time-of-flight mass spectrometer (LC–QTOF-MS), as reported previously [25]. Seven BAs were analyzed, with chole acid (CA), chenodeoxycholic acid (CDCA), and hyochlic acid (HCA) obtained as primary BAs and ursodeoxychlic acid (UDCA), deoxycholic acid (DCA), lithocolic acid (LCA), and 7-oxo-DCA as secondary BAs. Each BA item was analyzed in terms of concentrations and percentages of the total, with the percentage denoted in the form % primary BA, % secondary BA, etc.

### 2.4. SCFA Measurement

Samples for SCFA measurement were obtained by adding 10 mg of lyophilized fecal sample and ethanol (20 mg/mL) to internal standard in a tube with zirconia beads and subjecting to homogenization (8700 rpm, 15 s × 2, interval 5 s) and centrifugation (1600× *g*, 4 °C, 10 min). To the resulting supernatant (50 μL per sample) were then added 50 μL of 50 mM 3-nitrophenylhydrazine hydrochloride, 50 μL of 50 mM 1-ethyl-3-carbodiimide hydrochloride, 50 μL of 7.5% pyridine, and 50 μL of 75% methanol and these were mixed under shielded light. The mixture was then diluted 5-fold with 0.5% formic acid–75% methanol, and 50 μL injected into an LC–MS (SHIMADZU LCMS-8050, Kyoto, Japan) for analysis. The 4 fecal SCFAs, acetic acid, propionic acid, isobutyric acid, and butyric acid, were then measured quantitatively, and 11 other fecal SCFAs and metabolites, such as succinic acid and pyruvic acid, were measured in terms of relative area ratios due to the unavailability of standards.

### 2.5. DNA Extraction and 16S rRNA Gene Amplicon Sequencing

The fecal sample mixture was mechanically disrupted using the bead-beating method. DNA was extracted using the Gene Prep Star PI-80X (Kurabo Industries, Osaka, Japan). The V3–V4 region of the 16S rRNA gene was amplified via PCR using published primers [26], based on DNA extraction. Amplicons were sequenced via the paired-end method using MiSeq (Illumina, San Diego, CA, USA). The overall procedure, from fecal sampling to 16S rRNA sequencing, was performed according to a previously described protocol [27].

### 2.6. Bioinformatics Analysis

The paired-end output from MiSeq was trimmed and merged before selecting the operational taxonomic units (OTUs). OTU classification and diversity analysis were performed using the QIIME pipeline (v. 1.9.1) [28]. All steps from trimming to diversity analysis were performed automatically according to previously described methods [29]. The obtained OTUs were clustered against the SILVA 128 reference database [30] at 97% similarity using the USEARCH algorithm [31]. Taxonomic classification from the phylum to the genus level was performed using the SILVA 128 reference database. Bacterial names are prefixed with p_, o_, f_, and g_ to indicate phylum, order, family, and genus, respectively. All bacterial names are given in italics.

### 2.7. Statistical Analysis

The output of the QIIME pipeline in the Biom table format was imported and analyzed using R (version 3.5.1). The alpha diversity index was calculated using the *estimate_richness* function in the “phyloseq” R package, with dominant bacteria from the phylum to the genus level defined as those with an average bacterial composition of at least 0.5%. 

The sample size was calculated based on the median Shannon value (2.918), which is a commonly used alpha diversity index that was utilized in our pilot study of patients with SMID (*n* = 10). The Shannon value was thus divided into high and low levels. To achieve a power of 0.8 with a two-sided significance level of 0.05 and an effect size of 1.85 by using G*Power 3.1.9.4, six participants were required in each group; thus, to allow for a possible 10% dropout rate, 14 patients were recruited overall. The stool sample collection methods differed from that used in the pilot study. Data describing healthy subjects were obtained from the Japan Microbiome Database of National Institutes of Biomedical Innovation, Health, and Nutrition (NIBIOHN JMD), which uses the same sampling method as that used in this study; thus, the mean minus one standard deviation (3.571) was used as the cutoff value, representing the lower limit for approximately 70% of normal subjects in the relevant age group. Data on the gut microbiome of 94 healthy male and female samples under 30 years of age, including the age range of the patients in this study, were obtained as a healthy control group (HC group). Patients demonstrating values higher than the cutoff were grouped into the high-diversity group (HD group, *n* = 8), and those with results lower than the cutoff into the low-diversity group (LD group, *n* = 6). The Shannon’s power obtained via this grouping of 0.964 is higher than the 0.8 required.

The Wilcoxon rank sum test and Fisher’s exact test were used for comparison analysis between the two groups, the HD group and the LD group. The Kruskal–Wallis test was used for the comparison analysis between the three groups, including the HC group, and when a statistically significant difference was confirmed, the Dunn’s test and Bonferroni correction were performed, as well as Spearman correlation for correlation analysis. For covariates that were found to affect the results, analysis of covariance (ANCOVA) was performed. Statistical analyses were performed using the Statistics Premium Grad Pack Version 28 (IBM, Armonk, NY, USA). All statistical tests were two-sided, with significance or trend levels of *p* < 0.05 and *p* < 0.1, respectively. Data are shown as the median (minimum, maximum).

## 3. Results

### 3.1. Characteristics of the Patients Overall and Each Group

The overall characteristics of the patients are presented in Table 1. Height for age (H/A) was severely stunted (H/A: <85) in three patients, moderately stunted (H/A: 85–90) in three, and normal (H/A: 90–110) in eight; weight for height (W/H) was severely wasted (W/H: <70) in two, mildly wasted (W/H: 80–90) in four, normal (W/H: 90–110) in five, and overweight (W/H: >110) in three. Regarding motor function, seven patients were bedridden, one could sit with support, and six had gait disturbance, while in terms of nutritional intake methods, five patients were on oral intake, seven were on gastrostomy, and two received nutrition via a combination of oral and gastronomy methods. Six patients received only enteral formula containing dietary fiber, while the rest received normal food in mashed or paste form (*n* = 7) or a combination of normal food and enteral formula (*n* = 1). The types and amounts of oligosaccharides contained in enteral formula were as follows: patient No. 7: 0.5 g galacto-oligosaccharide with lactic acid bacteria (heat-killed *Enterococcus faecalis*), No. 10: 0.8 g galacto-oligosaccharide, No. 11: 1.0 g fructo-oligosaccharide, No. 13: 0.26 g soybean oligosaccharide. 

The diversity of the HD and LD groups is shown in Figure S1, in which data are compared with other diversity indices (Simpson, Observed, Fisher, and Chao1), including the Shannon index. Intergroup comparison of each diversity index showed that the HD group was comparable to the mean of the 94 healthy NIBIOHN JMD samples for the same age group as the patients in this study, while the LD group was lower.

The differences in the characteristics of the HD and LD groups are shown in Table 2. Although no significant differences were observed between the groups in terms of height or weight, the H/A was significantly lower in the LD group than in the HD group, which indicates long-term undernutrition and stunting (*p* = 0.029). On the other hand, no significant difference was observed for W/H, indicating short-term undernutrition (*p* = 0.181). A comparison of patients who were bedridden with those sitting with support or showing gait disturbance showed that all patients in the LD group were bedridden, with a significant difference between the two groups (*p* = 0.005). A comparison of weaning showed that the three patients who had never received weaning food belonged to the LD group, with a tendency for differences between the two groups observed (*p* = 0.055). When the main nutritional intake method, i.e., oral vs. gastrostomy, was compared, all patients in the LD group received nutrition via gastrostomy, and there was a significant difference between the two groups (*p* = 0.0097). Regarding the type of meals, all patients in the HD group received regular, mashed, or paste diets with or without enteral formula, while all patients in the LD group received only enteral formula, again indicating significant differences between the two groups (*p* < 0.001). The one bedridden patient (No. 2) and two gastrostomy patients (No. 1 and No. 8) in the HD group had the same physical characteristics as those in the LD group but received meals with or without enteral formula. This difference in the types of meals dichotomized the characteristics of the two groups in terms of different diversity. 

### 3.2. Comparisons Between Groups

#### 3.2.1. Nutrient Intake, Prescription Drugs, Blood Biochemical Tests, and Defecation Status

A comparison of the nutrient intake for each group is shown in Table S1. Energy, protein, and carbohydrate per kg of body weight were significantly lower in the LD group than the HD group (*p* = 0.029, 0.008, and 0.043, respectively). 

The 15 categories of prescription drugs were compared by the number of types taken and the number of patients taking them (Table S2). Muscle relaxants were taken by three patients, all of whom belonged to the LD group, with a trend of difference observed between the two groups (*p* = 0.055). No significant differences were observed between the two groups for the other categories of drugs.

A comparison of the results of the blood biochemical tests can be seen in Table S3. The two patients for whom blood collection could not be performed belonged to the LD group. Serum total protein (TP), albumin (Alb), and blood urea nitrogen (BUN) were significantly higher in the HD group than in the LD group (*p* = 0.048, 0.048, and 0.048, respectively), while platelets (PLT) were significantly lower in the HD group (*p* = 0.028). However, differences in the median values of the laboratory data were observed within the normal reference range. No significant differences were observed between the two groups for RTPs, such as prealbumin, retinol-binding protein, and transferrin, indicating no short-term undernutrition in either group.

In terms of defecation status (Figure S2), the CAS assessment of constipation symptoms indicated no significant difference between the two groups, with 0 (0, 1) points in the HD group and 0 (0, 1) points in the LD group (*p* = 0.573), and neither group showing constipation symptoms. However, significant differences were observed for BSS, which indicates stool shape, in the HD and LD groups, with results of 4.2 (2.0, 5.3) and 5.8 (5.1, 6.4), respectively (*p* = 0.003). Scores of 3–5 points indicate normal stools, <3 indicates hard stools, and >5 indicates loose stools [32]. Thus, the LD group had significantly looser stools.

#### 3.2.2. Gut Microbiome

**Figure 1:**
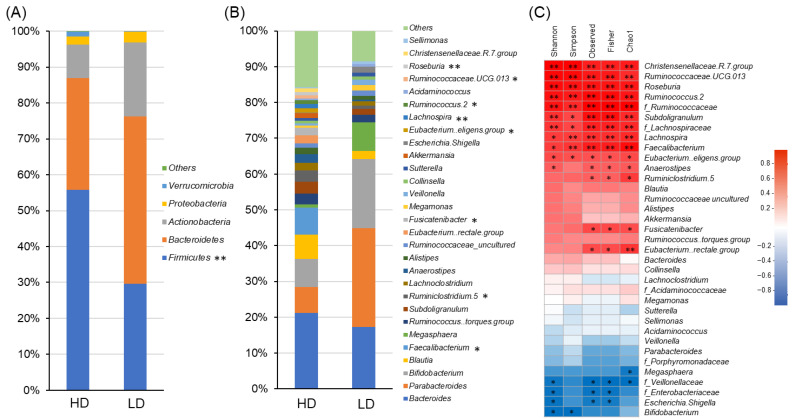
Comparison of gut microbiome for each group and correlation between alpha diversity and gut microbiome. Comparison of the (**A**) phylum and (**B**) genus in each group was obtained via Mann–Whitney *U* test. (**C**) Correlation between alfpha diversity and genus/family by Spearman. ** *p* < 0.01. * *p* < 0.05.

Intergroup comparisons of the gut microbiome (Figure 1 and Table S4) indicated that, at the phylum level, the occupancy of *Firmicutes* was significantly higher in the HD group than in the LD group (*p* = 0.008; Figure 1A). The HD group had a significantly higher presence of genera belonging to *p_Firmicutes*, such as *Eubacterium eligens group*, *Fusicatenibacter*, *Lachnospira*, *Roseburia*, *Ruminococcus 2*, *Feacalibacterium*, *Ruminiclostridium 5*, and *Ruminococcaceae UCG 013* (at *p* = 0.020, 0.043, 0.005, 0.005, 0.020, 0.013, 0.043, and 0.029, respectively; Figure 1B). Correlations between the alpha diversity and genus and family level of the gut microbiome can be seen in Figure 1C. All diversity indices—Shannon, Simpson, Observed, Fisher, and Chao1—correlated with a nearly identical gut microbiome overall, indicating significant positive correlations between diversity indices and genera belonging to *p_Firmicutes*. This result is similar to the significant differences observed for several of the observed bacteria from genera belonging to the *f_Ruminococcaceae* and *f_Lachnosperaceae*, with significant positive correlations. However, *g_Bifidobacterium*, *g_Escherichia–Shigella*, which belongs to the *f_Enterobacteriaceae*, and *f_Veillonellaceae* showed significant negative correlations with some of the diversity indices, such as the Shannon index.

The results of the three-group comparison, which included the HC group, are shown in Figure S3 and Table S4, and significant differences were observed not only in the *p_Firmicutes* but also in the *p_Bacteroidetes* and *p_Actinobacteria* (*p* = 0.004, 0.041, and 0.009, respectively). Post hoc test results showed that the *p_Firmicutes* tended to be lower in the LD group than in the HC group (*p* = 0.085), and *p_Actinobacteria* was significantly higher (*p* = 0.030). In addition, *f_Lachinospiraceae* and *f_Ruminococcaceae* were significantly lower (*p* = 0.006 and 0.047, respectively), *g_Bifidobacterium* was significantly higher (*p* = 0.018), and *f_Enterobacteriacae* tended to be high (*p* = 0.099), indicating that the gut microbiome composition was significantly different from that of the HC group. On the other hand, the *p_Firmicutes* tended to be higher in the HD group than in the HC group (*p* = 0.051), and *p_Bacteroidetes* was significantly lower (*p* = 0.040), but there was no significant difference between the HD and HC groups for the above bacteria that showed differences between the LD and HC groups. The HD group had a similar diversity index to the HC group, but the gut microbiome composition was different.

#### 3.2.3. Fecal Metabolites (BAs and SCFAs)

**Figure 2:**
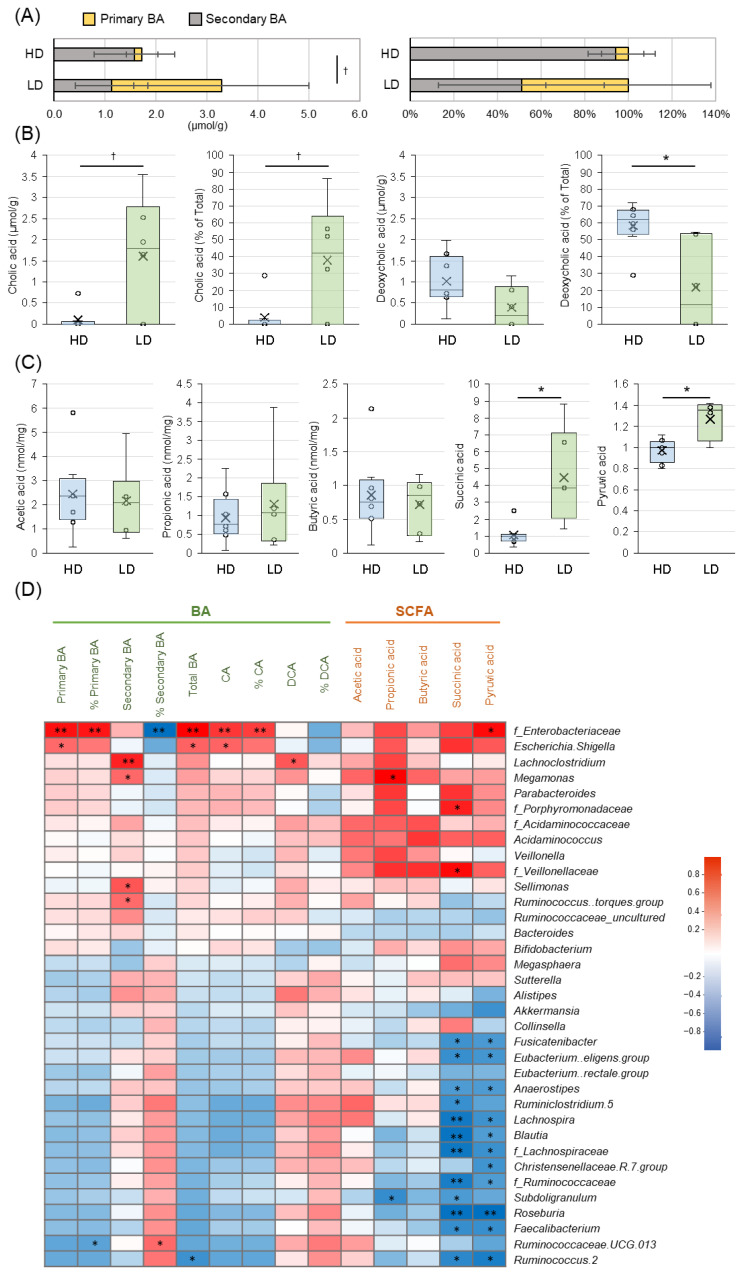
Comparison of fecal metabolites (BAs and SCFAs) in each group and correlation between fecal metabolites and the gut microbiome. (**A**) Comparison of primary and secondary BAs in the two groups. Comparison is by quantity on the left and the percentage of each BA to total BA on the right. Mean ± SD. (**B**) Comparisons of cholic acid (CA; quantity and percentage) as a primary BA and deoxycholic acid (DCA; quantity and percentage) as a secondary BA are shown. Median (IQR). (**C**) Comparison between groups of SCFAs. Acetic acid, propionic acid, and butyric acid are shown as concentrations, and succinic acid and pyruvic acid as relative area ratios with the median value of the HD group as 1. Median (IQR). (**A**–**C**): Mann–Whitney *U* test. (**D**) Correlation between BAs/SCFAs and genus/family by Spearman. ** *p* < 0.01. * *p* < 0.05. ^†^ *p* < 0.1.

Intergroup comparisons of the fecal metabolites, BAs and SCFAs, are shown in Figure 2. Total BAs of 1.8 (0.3, 3.0) μmol/g were obtained from feces in the HD group and 3.8 (1.1, 5.0) μmol/g in the LD group, indicating higher levels in the LD group as compared to the HD group (*p* = 0.081, left side of Figure 2A; figures show mean ± SD, and Table S5). The HD group showed almost no primary BA and was dominated by secondary BA, whereas the LD group included more primary BA. In terms of percentage of the total, the HD group included 0 (0, 37.0)% primary BA and 100 (63.0, 100)% secondary BA, while the LD group had 56.5 (0, 92.6)% primary BA and 43.5 (6.9, 100)% secondary BA, indicating that the composition of primary and secondary BAs differed in the two groups (right side of Figure 2A). In particular, in terms of primary BAs, CA tended to be higher in the LD group in terms of both quantity and percentage of the total BA (*p* = 0.081, 0.081, respectively; Figure 2B). For secondary BAs, DCA was significantly lower in the LD group than in the HD group as a percentage of total BA (*p* = 0.020). However, no significant difference was observed between the two groups in terms of quantity (*p* = 0.108). In terms of the correlation between BAs and the gut microbiome (Figure 2D), *f_Enterobacteriaceae* showed significant positive correlation with primary BA, including CA, and total BA, and significant negative correlation with % secondary BA (*p* < 0.01, respectively), while secondary BA showed significant positive correlations with *g_Lachinoclostridium*, *g_Sellimonas*, and *g_Ruminococcus torques* group, belonging to *f_Lachinospiraceae* and *g_Ruminococcaceae UCG013*, which in turn belong to *f_Ruminococcaceae* and *g_Megamonas* (*p* < 0.05, respectively). 

A comparison of the SCFAs and other metabolites in the two groups is shown in Figure 2C and Table S6. No significant differences were observed in quantitative measurements of acetic acid, propionic acid, and butyric acid for the groups (*p* = 0.491, 0.755, 1.000, respectively); however, succinic acid and pyruvic acid were significantly higher in the LD group than the HD group by relative value (*p* = 0.003, 0.008, respectively). Correlation analysis of the SCFAs and the gut microbiome (Figure 2D) indicated significant negative correlations between succinic acid and pyruvic acid and *f_Lachinospiraceae* and *f_Ruminococcaceae*, and both metabolites showed significant negative correlations with similar bacteria, such as *g_Roseburia* and *g_Feacalibacterium*, and others belonging to these families.

#### 3.2.4. Covariates Adjustment

Significant differences between the two groups were observed in terms of physical severity factors such as motor function, main nutritional intake method, experience of weaning food, and muscle relaxant medication, as well as nutrient intake in terms of energy, protein, and carbohydrates per kg of body weight. Using these as covariates, Table S7 shows the results of ANCOVA analysis of defecation status, nutritional status, and fecal metabolites, with significant differences observed between the two groups. Significant differences or significant trends were observed between the HD and LD groups in terms of BSS and % DCA without the influence of all covariate items, indicating that high and low diversity affect BSS and % DCA. ANCOVA was also performed for each diversity index. The Observed, Fisher, and Chao1 indices indicating species richness [33], were found to be affected by motor function and the main nutrient intake method, while the Shannon and Simpson indices, indicating the richness and evenness of species comprising the microbiome [34], were unaffected by all covariates. 

## 4. Discussion

The objective of this study was to examine the association between gut microbiome composition and the physical characteristics of patients with SMID, with a focus on differences in gut microbiome diversity. Categorization, which was based on diversity data from healthy Japanese individuals under 30 years of age, indicated a similar level of diversity between the HD group and the HC group, whereas the LD group showed significantly lower diversity. The gut microbiome composition in the LD group strongly reflected the characteristics found in the neonatal and lactation stages, implying immaturity for the gut microbiome in the LD group. The LD group also had a significantly lower % DCA in their fecal metabolite BAs, indicating potential insufficiency in the conversion of primary BAs to secondary BAs. All members of the LD group were bedridden and received only enteral formula via gastrostomy, including three patients who had never been given weaning food and three patients who were taking muscle relaxants. The LD group, therefore, demonstrated greater physical severity than the HD group. Moreover, the composition of the gut microbiome and BAs in the gut of the LD group differed from those of the HC and HD groups. This study is the first to suggest an immature gut environment for such patients.

Diversity data from healthy Japanese individuals under 30 years of age were utilized for group classification. The cutoff value was set at the mean minus one standard deviation, serving as the lower threshold applicable to approximately 70% of healthy subjects. While the diversity in the HD group was comparable to the mean value of the HC group, the diversity in the LD group was significantly lower (Figure S1). Comparison of the gut microbiome composition in the groups revealed a marked reduction in *p_Firmicutes* for the LD group as compared to the HD group, along with significantly lower levels of *f_Lachnospiraceae* and *f_Ruminococcaceae* and several other genera within these families (Figure 1, Table S4). Conversely, *g_Escherichia–Shigella* showed a tendency for elevation. Alpha diversity was found to have a significant positive correlation with *f_Lachnospiraceae*, *f_Ruminococcaceae*, and some of the other genera in these families and a significant negative correlation with *g_Bifidobacterium*, *f_Enterobacteriaceae*, and *g_Escherichia–Shigella*. In the comparison of the three groups, including the HC group (Figure S3, Table S4), the LD group had significantly lower values for *f_Lachinospiraceae* and *f_Ruminococcaceae*, and higher values for *g_Bifidobacterium* and *f_Enterobacteriacae*, than the HC group. It is known that the human gut microbiome transitions from dominancy of *f_Enterobacteriaceae* and *g_Bifidobacterium*—typical during neonatal and lactation periods—to that of *p_Firmicutes* bacteria under the introduction of weaning foods containing dietary fiber, accompanied by an increase in diversity [7]. Notably, the taxa *g_Roseburia*, *g_Faecalibacterium*, and *g_Subdoligranulum*, which were particularly low in the LD group, are known to increase with gut microbiome maturation [35]. The gut microbiome observed in the LD group in this study shares similarities with the gut microbiome characteristics observed during the neonatal and lactation stages in previous studies, indicating that the gut microbiome in the LD group may still be immature. On the other hand, the *p_Firmicutes* tended to be higher in the HD group than in the HC group, while *p_Bacteroidetes* was significantly lower. The above bacteria, which indicated differences in the characteristics of gut microbiome in the neonatal and lactation period between the LD and HC groups, did not show significant differences between the HD and HC groups. The HD group showed a diversity index similar to that of the HC group, but there were differences in the composition of the gut microbiome, and one of the factors considered to be responsible for this was the difference in age group.

In the analysis of fecal metabolites, no significant differences were observed between the two groups in terms of SCFAs, such as acetic acid, propionic acid, and butyric acid. However, metabolites such as succinic acid and pyruvic acid, which are intermediate metabolites of the citric acid cycle and SCFA precursors [36], were significantly elevated in the LD group as compared to the HD group (*p* = 0.003 and 0.008, respectively) and exhibited negative correlations with *f_Lachnospiraceae* and *f_Ruminococcaceae*, as well as with *g_Roseburia* and *g_Faecalibacterium*, which are in the same families. A previous study found that critically ill children in a pediatric intensive care unit (PICU) had lower levels of *g_Roseburia* and *g_Faecalibacterium* as compared to healthy children, which was linked to the accumulation of intermediate metabolites, including succinic acid and pyruvic acid [36]. The observed association between these metabolites and the gut microbiome composition in the LD group is consistent with earlier findings, suggesting that the LD group may suffer from inadequate conversion of succinic acid and pyruvic acid to acetic acid, propionic acid, and butyric acid. However, the use of ANCOVA with physical severity (including motor function, nutritional intake method, experience with weaning foods, and the use of muscle relaxants) and dietary intake (energy, protein, and carbohydrate intake) as covariates indicated no significant differences between the two groups for these two SCFAs in terms of motor function and nutritional intake methods (Table S7), implying that the accumulation of SCFA intermediate metabolites in the LD group may be influenced not only by the level of gut microbiome diversity but also by factors such as physical severity, including motor function and the nutritional intake method. SCFAs are a major energy source for the colonic mucosa and a non-negligible energy substrate for systemic energy and fat metabolism [37]. In addition, by acting on immune cells, they show anti-inflammatory effects and promote the production of IgA antibodies in the intestinal tract, playing an important role in immune regulation, such as protection against infection [38]. Increasing the maturity of the gut microbiota may promote the conversion of intermediate metabolites such as succinic acid and pyruvic acid into SCFAs, and this may lead to improvements in intestinal health, immune regulation, and nutritional status.

In terms of fecal metabolic BA products, the LD group exhibited a trend toward higher total BA levels as compared to the HD group (*p* = 0.081), with observable differences in the composition of primary and secondary BAs noted in the two groups (Figure 2A). Primary BA is synthesized in the liver and secreted into the intestinal tract for fat absorption, after which it is primarily reabsorbed or travels throughout the body. Unabsorbed BA is metabolized by intestinal bacteria and converted to secondary BA. Secondary BA strongly activates the receptor TGR5, which is involved in a wide range of physiological functions, including glucose, lipid, and energy metabolism, regulating the immune response, intestinal motility, and water secretion [10,11]. Primary BA accounted for a smaller proportion of the total in the HD group, with secondary BA composing most of the total, whereas nearly half of the BA in the HD group was primary. Specifically, CA and % CA levels were significantly elevated in the LD group (*p* = 0.081 for both), while % DCA, a secondary BA, was significantly lower (*p* = 0.020). Correlation analysis between BAs and the gut microbiome revealed a positive correlation between *f_Enterobacteriaceae* and the total and primary BAs and a negative correlation with secondary BAs. Conversely, certain genera within *f_Lachnospiraceae* and *f_Ruminococcaceae*, such as *g_Lachnoclostridium*, *g_Ruminococcus torques* group, and *g_Ruminococcus UCG013*, showed a positive correlation with the secondary BAs. Even after adjusting for physical severity and dietary status using ANCOVA, the % DCA between the two groups remained significantly different across all covariates, indicating that the secondary BA levels are influenced by the diversity of the gut microbiome (Table S7).

It is known that the composition of BA evolves during infancy as the gut microbiome matures. The major BAs in stools during the first three years of life transition from conjugated BAs to primary BAs, then to urso-type BAs, and finally to secondary BAs [11]. Primary BA is thus predominant before weaning; however, the proportion of secondary BA increases as weaning begins and the gut microbiome matures [11]. CA is converted to DCA by 7α-dehydroxylation, a process involving eight intracellular enzymes that are encoded by the bile-acid-inducible (bai) gene cluster in bacteria [39,40]. This bai cluster is most commonly found in *f_Ruminococcaceae*, with some bacteria in *f_Lachnospiraceae* also playing a significant role [41]. Dehydroxylation is also associated with gut microbiome diversity [11]. Secondary BAs make up more than 90% of the BA composition in about 85% of adults [42]; thus, the high prevalence of secondary BAs observed in the HD group is similar to the BA composition typically seen in adults. Conversely, the BA composition in the LD group, where primary BAs were more dominant, resembles that of infancy. The immaturity of the gut microbiome in the LD group may be at least partially due to the lower numbers of *f_Ruminococcaceae* and *f_Lachnospiraceae*, which play an important role in the conversion to secondary BAs, and the enhanced *f_Enterobacteriaceae*. BAs play a role in the energy, lipid, and glucose metabolism via BA receptors [42]. In mouse experiments, it has been reported that when the gut microbiota is artificially disrupted in a way that prevents the production of secondary BAs by antibiotics, blood glucose levels and triglyceride concentrations decrease, indicating that secondary BAs play an important role in regulating blood glucose levels and blood lipid concentrations [43]. Although the blood tests in this study did not show any significant difference in blood glucose levels or blood lipids between the two groups, the immaturity of the BA composition in the LD group may be affecting the nutritional status, such as stunting. It is not known how the changes in gut bacterial colonization and BA composition seen in infancy affect the maturation of a host’s metabolic and immune systems [42]. However, since changes in the BA composition are suspected to have a unique effect on the host physiology at each developmental stage [42], it is considered that promoting the maturation of the BA composition may contribute to improvements in acquired physical conditions such as peristalsis and nutritional status that are exhibited by patients with SMID.

Many patients with SMID experience constipation, as evidenced by our previous study involving 10 home care patients with SMID (median age 15.5 years, range 11–28), in which 60% exhibited constipation symptoms [5]. However, constipation symptoms were less prevalent in the current study, with no significant difference observed in the CAS for the two groups (Figure S2). Factors such as differences in the care environment (home care versus hospitalization), variations in the treatment used for constipation, and differences in the age range of the subjects might have influenced these results. The LD group demonstrated significantly higher BSS values than the HD group, indicating looser stools (*p* = 0.003). Even after adjusting for physical severity and dietary status using ANCOVA, significant differences in the BSS persisted between the two groups across all covariates, suggesting that stool consistency is influenced by gut microbiome diversity (Table S7). Previous studies have indicated that *f_Enterobacteriaceae* and *g_Escherichia–Shigella* can induce diarrhea [44], and that children with diarrhea tend to have lower gut microbiome diversity as compared to healthy children [45]. It has also been reported that an excess of total BAs reaching the large intestine can lead to diarrhea [46]. One commonly used drug, elobixibat, although none of the patients in this study were taking it, treats chronic constipation by increasing the amount of BA in the colon [47]. The drug works by inhibiting the ileal bile acid transporter (IBAT), which is expressed on epithelial cells in the terminal ileum, thereby reducing the BA reabsorption and augmenting the flow of BAs into the colonic lumen. The process stimulates water secretion in the colonic lumen, alleviating constipation [47]. Succinic acid, which is absorbed slowly in the large intestine, is known for its high water retention capacity and can increase the stool water content, potentially contributing to diarrhea symptoms [48]. The LD group showed a tendency toward higher levels of *g_Escherichia–Shigella* and reduced diversity. Furthermore, elevated levels of total BAs and succinic acid in this group suggest that these factors may have contributed to the softer stools. 

In the LD group, the relative abundance of *g_Escherichia–Shigella* was high, considered to be one of the causes of softer stools. However, *g_Escherichia–Shigella* is one of the important pathogenic bacteria that causes diarrhea [44]; therefore, we examined the effects of taking antibiotics as one of the conditions that significantly affect the gut microbiome, including *g_Escherichia–Shigella*. One patient in the HD group who was taking small-dose long-term macrolide therapy was taking clarithromycin. Although there has been a report that taking clarithromycin in general slightly reduces the number of *f_Enterobacteriaceae*, to which *g_Escherichia–Shigella* belongs [49], there have been no reports of the effects of small-dose long-term clarithromycin therapy on *g_Escherichia–Shigella*. There was also one patient in the LD group who was taking small-dose long-term ST combination therapy, but it has been reported that this therapy may selectively suppress the growth of *o_Enterobacteriales*, to which *g_Escherichia–Shigella* belongs [23]. Although it is possible that both clarithromycin and the ST combination drug may have some effect on *g_Escherichia–Shigella*, as there was no significant difference between the two groups in the proportion of patients using these drugs, it is considered that the effect on *g_Escherichia–Shigella* is unlikely to have affected the other results, including softer stools. In addition, regarding the conditions that alter the gut microbiome, there was no significant difference in dietary fiber intake between the two groups (*p* = 0.852, Table S1). As for the intake of oligosaccharides and probiotics other than food, soybean oligosaccharides, fructo-oligosaccharides, and galacto-oligosaccharides were included in the enteral nutrition of four of the patients in the LD group. These oligosaccharides have been reported to affect gut bacteria such as *Bifidobacterium* [50], and while many oligosaccharides are not utilized by *E. coli*, galacto-oligosaccharides have been reported to promote the growth of *E. coli* when consumed in large quantities [50]. On the other hand, one patient who consumed galacto-oligosaccharides was also taking lactobacillus (*Enterococcus faecalis*). *E. faecalis* is included in some intestinal medicines, but there have been no reports of a link to *g_Escherichia–Shigella*. In previous studies that investigated the effects of oligosaccharides on gut microbiome, the daily doses used were much higher than those used in the LD group: 10 g of soybean oligosaccharides [50], 8 g of fructo-oligosaccharides [50,51], and 2.5 g and 5 g of galacto-oligosaccharides [51]. The low intake of oligosaccharides in the LD group is considered unlikely to have an effect on *g_Escherichia–Shigella*. The other condition that may have altered the gut microbiome is the fact that *g_Escherichia-Shigella* is an aerobic symbiotic bacterium that can proliferate even in the presence of oxygen, but in this study, it was not always possible to process samples immediately after defecation because all patients, with the exception of one in the HD group, defecated into diapers. If this bacterium is to be the focus of future research, it will be necessary to revise the research design to enable a strict comparison of the amount of *g_Escherichia-Shigella*.

H/A, which reflects long-term undernutrition along with the markers of nutritional status, TP, ALB, and BUN, was significantly lower in the blood biochemistry of the LD group as compared to the HD group (Table 2 and Table S3). One study comparing the gut microbiome of malnourished Bangladeshi children with acute malnutrition and stunting with that of healthy children revealed significant differences in the relative proportions of all four of the major phyla: *Firmicutes*, *Bacteroidetes*, *Actinobacteria*, and *Proteobacteria* [52]. Additionally, the malnourished children exhibited lower microbial diversity as compared to their healthy counterparts. The low nutritional status observed in the LD group was consistent with some of these gut bacteria and microbial diversity characteristics, which were shown to be associated in this previous study [52]. However, when ANCOVA was conducted with physical severity and dietary status as covariates, the differences in these nutritional status markers between the two groups were no longer significant (Table S7). This suggests that, while the LD group exhibits microbiome characteristics that are partially consistent with those seen in malnourished children, both the gut microbiome composition, including diversity, and factors such as primary disease and dietary status contribute to the nutritional status observed in the LD group. It has been reported that immature gut microbiome can cause a decrease in digestive function, leading to insufficient breakdown and absorption of nutrients, as well as a decrease in immune function and abnormal inflammatory reactions, which can increase the risk of infection and cause chronic inflammatory conditions [53,54,55,56]. These can result in nutritional deficiencies and weight loss. Although the inflammatory markers CRP, TNF-α, and IL-6 in the LD group were within the normal range, the decrease in nutritional status and stunting in the LD group suggest that the immaturity of the gut microbiome may affect the nutritional status of the patients with SMID. In addition, it is conceivable that the immaturity of the gut microbiome may lead to a lack of cross-talk between the host and the gut microbiome. There are few reports on the dynamic changes in the gut microbiota during infancy, but the mind and body of the host, the human, rapidly develop during this period. It is possible that gut bacteria contribute to normal human growth through interactions with the gut microbiota and the host. For example, it has been reported that the toxin of *Pasteurella multocida*, a common bacteria in pigs, affects the secretion of growth hormones in the host pig [57]. Therefore, improving the diversity of the gut microbiome and promoting maturation in the treatment of SMID patients may contribute to “more appropriate growth”, “prevention of chronic diseases”, and “comprehensive health management”. 

Regarding the physical characteristics, all individuals in the LD group were bedridden and relied exclusively on an enteral formula administered via gastrostomy. The LD group exhibited greater physical severity as compared to the HD group (Table 2). Previous research comparing the gut microbiome of patients with SMID that were tube-fed with that of healthy children reported dysbiosis and altered diversity in the SMID patients, with low dietary fiber intake being a potential contributing factor [14]. In the present study, eight of the fourteen SMID patients primarily received nutrition through tube feeding (gastrostomy). Of these, six belonged to the LD group, while two were in the HD group and demonstrated diversity levels comparable to healthy adults. The primary distinction between the two groups was significantly associated with whether patients received only enteral formula or a diet with varied ingredients, such as a softened regular meal in paste form (*p* < 0.001), and no differences in dietary fiber intake were observed. This suggests that the decrease in diversity is not directly caused by tube feeding itself but rather by the exclusive intake of enteral formula with specific ingredients. Nakai et al. [14] point out the need for further interventional studies involving the administration of prebiotics, probiotics, and synbiotics, to improve dysbiosis in children with SMID. It would be helpful to investigate the possibility of altering the gut microbiota and enhancing its diversity by using prebiotics, probiotics, and synbiotics, as well as incorporating varied meals alongside enteral formula in the diet of SMID patients. 

One limitation of this study is that it was conducted exclusively with hospitalized patients suffering from SMID at a single facility, which prevents comparisons with SMID patients at other disabled institutions or in home environments. Consequently, it is unclear whether the findings are unique to hospitalized SMID patients at this facility or applicable to all SMID patients. In addition, given the variability in the content and mode of consumption of the patients’ meals, a key future challenge will be to assess and compare the overall gut microbiome, its metabolites, and the nutritional status of SMID patients under standardized conditions, such as controlled dietary interventions. Moreover, the hospital-based defecation management of the subjects in this study was meticulous in that it allowed little examination of constipation symptoms, a significant important issue for patients with SMID. In this study, the Shannon index was utilized for diversity grouping. Like the Simpson index, Shannon accounts for both species richness and evenness [34]. ANCOVA, when adjusted for physical severity and dietary status, revealed significant differences in the Shannon and Simpson indices between the two groups for all covariates. Conversely, no significant differences were observed for the Observed, Fisher, and Chao1 diversity indices, which were utilized to measure species richness in the diversity index with regards to the motor function and nutritional intake method covariates when using ANCOVA [33]. As seen in Figure S1, significant differences were observed for the HD and LD groups (*p* < 0.01) in terms of all diversity indices. However, some may have been influenced by motor function and dietary intake methods. Despite these limitations, this is the first study to demonstrate that BAs and other metabolites in the guts of some SMID patients are characterized by low microbial diversity (the LD group) and differ from those in healthy individuals (the HC group) and SMID patients in the HD group, suggesting an immature gut environment. Furthermore, the results suggest that the differences in diversity may be influenced by acquired physical conditions such as stool shape and nutritional status, in addition to the physical severity influenced by underlying diseases such as motor function impairment, nutritional intake methods, and muscle relaxant use. We hope that the findings of this study will bring a renewed focus on approaches to increasing diversity in SMID patients with low diversity and lead to improved maturation of the gut microbiota and metabolite composition, as well as better management of acquired somatic symptoms. 

## 5. Conclusions

This study explored the association between gut microbiome composition and physical characteristics in patients with SMID, focusing on differences in microbial diversity. The findings revealed that SMID patients with lowered microbial diversity exhibited greater physical severity and exclusively consumed enteral formula. The composition of the gut microbiome and BAs in the gut differed from those of healthy individuals and the high-diversity group, indicating a potentially immature gut environment. 

## Figures and Tables

**Table 1:** Characteristics of the patients.

	^1^ Sex	Age (Year)	^2^ Ht (m)	^2^ Wt (kg)	^3^ H/A (%)	^3^ W/H (%)	^4^ Motor Function	^5^ Intake Method	Meal Details	Weaning Experience	Diagnosis	^6^ Group
1	M	6	1.03	16.2	91.5	101.3	Gait disturbance	Gastro (Oral)	Paste diet	No	Cerebral palsy, Autism spectrum	HD
2	M	6	1.15	17.5	101.8	85.4	Bedridden	Oral (Gasto)	Mashed diet	Yes	Cerebral palsy, Intractable epilepsy	HD
3	M	7	1.21	20.0	101.7	87.0	Bedridden	Gastro	Enteral formula	No	Sequelae of hypoxic encephalopathy, Epilepsy	LD
4	M	9	1.20	28.3	92.3	125.8	Gait disturbance	Oral	Mashed diet	Yes	Fukuyama congenital muscular dystrophy	HD
5	M	11	1.12	24.0	78.9	126.3	Bedridden	Gastro	Enteral formula	Yes	Cerebral palsy, Microcephaly, Epilepsy	LD
6	M	12	1.41	33.8	94.6	98.0	Gait disturbance	Oral	Mashed diet	Yes	Symptomatic epilepsy	HD
7	F	12	1.28	22.2	85.3	85.4	Bedridden	Gastro	Enteral formula	No	Cerebral atrophy, Infantile spasms	LD
8	F	13	1.48	32.0	96.1	80.0	Sit with support	Gastro	Enteral formula and paste diet	Yes	Epilepsy sequelae	HD
9	M	13	1.38	35.1	87.9	106.4	Gait disturbance	Oral	Regular diet	Yes	Cerebral palsy, Periventricular Leukomalacia	HD
10	F	14	1.18	21.1	75.6	95.9	Bedridden	Gastro	Enteral formula	Yes	Sequelae of acute encephalopathy	LD
11	F	14	1.35	19.9	86.5	66.3	Bedridden	Gastro	Enteral formula	Yes	Dentato-ruburo- pallido-luysian atrophy, Epilepsy	LD
12	F	14	1.46	50.3	93.3	132.4	Gait disturbance	Oral	Regular diet	Yes	Cerebral palsy, Extremely low birth weight	HD
13	M	15	1.42	22.5	84.8	63.4	Bedridden	Gastro	Enteral formula	No	Sequelae of hypoxic encephalopathy	LD
14	F	17	1.45	36.0	92.1	97.3	Gait disturbance	Oral	Regular diet	Yes	Cerebral palsy, Epilepsy, Extremely low birth weight	HD

^1^ M, male; F, female. ^2^ Ht, height; Wt, weight. ^3^ H/A, height for age; W/H, weight for height. ^4^ Motor function is based on Oshima’s classification. ^5^ Oral, oral feeding; Gastro, gastronomy. When listed side by side with a method in parentheses, the one not in parentheses is the main intake method. ^6^ Groups with high and low diversity of gut microbiome. HD: high-diversity group; LD: low-diversity group.

**Table 2:** Differences in the characteristics of each group.

Item		HD Group	LD Group	*p*-Value
Number (men)		8 (5)	6 (3)	^a^ 1.000
Age (year)		12.5 (6, 17)	13.0 (7, 15)	^b^ 0.573
Height (m)		1.40 (1.03, 1.48)	1.25 (1.12, 1.42)	^b^ 0.414
Weight (kg)		32.9 (16.2, 50.3)	21.7 (19.9, 24.0)	^b^ 0.142
Height for age (H/A) (%)		92.8 (87.9, 101.8)	85.1 (75.6, 101.7)	^b^ 0.029 *
Weight for height (W/H) (%)		99.6 (80.0, 132.4)	86.2 (63.4, 126.3)	^b^ 0.181
Motor function (*n*)	Bedridden	1	6	^a^ 0.005 **
	Sit with support or gait disturbance	7	0	
Experience of weaning food (*n*)	Yes	8	3	^a^ 0.055 ^†^
	No	0	3	
Main nutritional intake method (*n*)	Oral	6	0	^a^ 0.0097 **
	Gastronomy	2	6	
Types of meals (*n*)	Meals with or without enteral formula (regular, mashed, paste)	8	0	^a^ < 0.001 **
	Enteral formula only	0	6	

Data are shown as median (minimum, maximum). ^a^ Mann–Whitney *U* test. ^b^ Fisher’s exact test. ** *p* < 0.01. * *p* < 0.05. ^†^ *p* < 0.1.

## Data Availability

The dataset used for the analysis in the current study is available from the corresponding author on reasonable request.

## Supplementary Materials

The following supporting information can be downloaded at: https://www.mdpi.com/article/10.3390/nu16203546/s1, Figure S1: Comparison of alpha diversity between the HD group and the LD group; Figure S2: Comparison of defecation status between the HD group and the LD group; Figure S3: Comparison of gut microbiome in the HC, HD, and LD group; Table S1: Comparison of nutrient intake for each group; Table S2: Comparison of prescription drugs for each group; Table S3: Comparison of blood biochemical tests for each group; Table S4: Comparison of gut microbiome for each group; Table S5: Comparison of BAs for each group; Table S6: Comparison of SCFAs for each group; Table S7: ANCOVA *p*-value excluding the effect of covariates for items with significant group differences between the HD group and the LD group.
