# Supplementary material for: Association Between Gut Microbiome Composition and Physical Characteristics in Patients with Severe Motor and Intellectual Disabilities: Perspectives from Microbial Diversity
Source: Nutrients. 2024 Oct 19;16(20):3546. doi: 10.3390/nu16203546 (PMC11510305; doi:10.3390/nu16203546)
Supplement: Supplementary file 1 [file nutrients-16-03546-s001.zip › nutrients-3242669-supplementary.pdf]

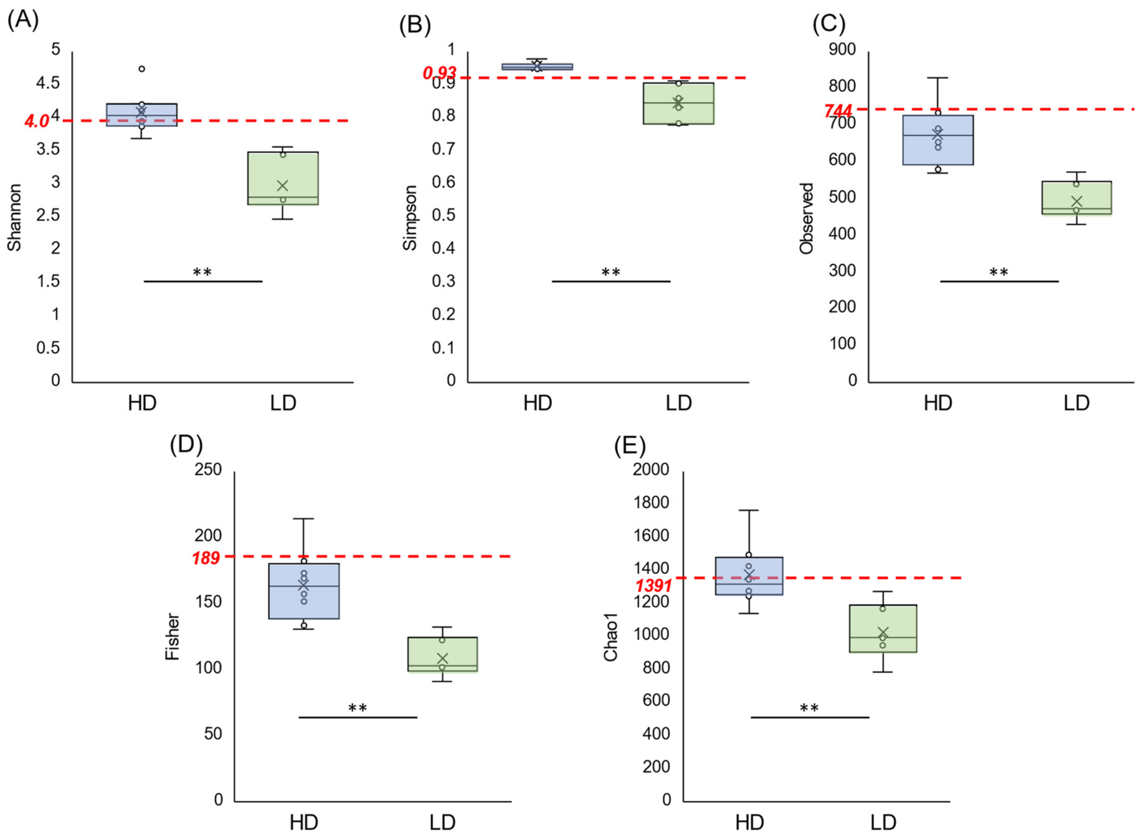

Figure S1. Comparison of alpha diversity between the HD group and the LD group. **(A)** Shannon. **(B)** Simpson. **(C)** Observed. **(D)** Fisher. **(E)** Chao1. The red dotted lines and values are the mean values in 94 healthy male and female samples under 30 years of age in the Japan Microbiome Database of National Institutes of Biomedical Innovation, Health and Nutrition (NIBIOHN JMD). Median (IQR). Mann-Whitney *U* test. ** $p < 0.01$.

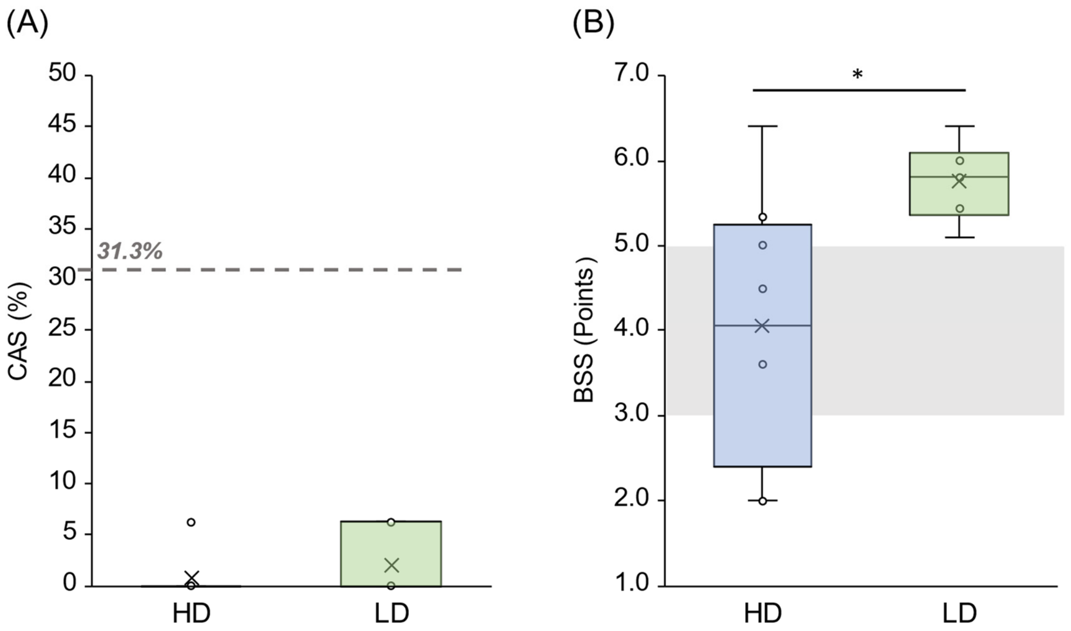

Figure S2. Comparison of defecation status between the HD group and the LD group. **(A)** CAS, constipation assessment scale. The percentage of people with constipation is ≥ 31.3%. **(B)** BSS, Bristol Stool Scale. A score of 3 to 5 points, indicated by a gray bar, indicates normal stools, a score of less than 3 points indicates hard stools, and a score of 5 or more indicates soft stools. Median (IQR). Mann-Whitney *U* test. * $p < 0.05$.

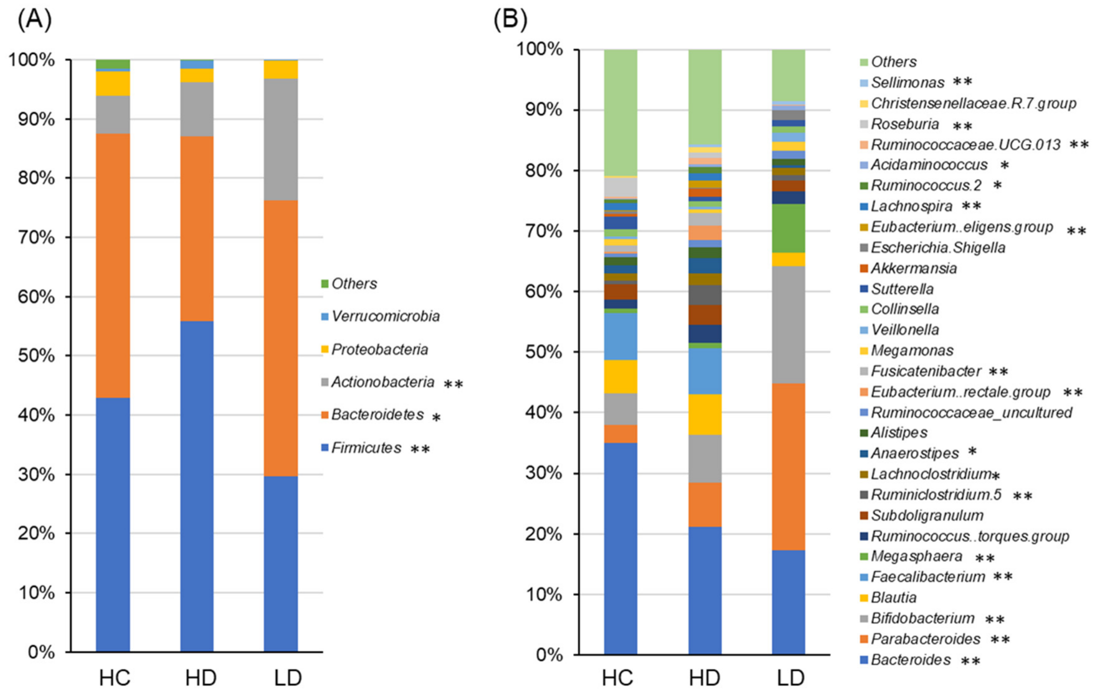

Figure S3. Comparison of gut microbiome in the HC, HD, and LD group. **(A)** phylum and **(B)** genus. Kruskal-Wallis test. When a statistically significant difference was confirmed, the Dunn's test and Bonferroni correction were performed. ** $p < 0.01$, * $p < 0.05$ (Kruskal-Wallis test).
